# The complete chloroplast genome sequence of *Nicotiana debneyi* (Solanaceae)

**DOI:** 10.1080/23802359.2021.1899074

**Published:** 2021-03-19

**Authors:** Jianmin Zeng, Changjun Huang, Yong Liu, Cheng Yuan, Haiqin Yu, Biqing Song

**Affiliations:** a Yunnan Academy of Tobacco Agricultural Sciences, Kunming, P.R. China; b Yuxi Zhongyan Tobacco Seed Co., Ltd, Yuxi, P.R. China

**Keywords:** Chloroplast genome, *Nicotiana debneyi*, phylogenetic analysis

## Abstract

In present study, we report the complete chloroplast genome of *Nicotiana debneyi*, a species endemic to eastern coast of Australia. The total genome size of *N.debneyi* is 156,073 bp in length, containing a large single-copy region of 86,672 bp, a small single-copy region of 18,581 bp, and two inverted repeat regions of 25,410 bp. The all GC content of *N.debneyi* chloroplast genome is 38.4%. It encodes a total of 129 unique genes, including 84 protein-coding genes, 37 tRNA genes, and eight rRNA genes, of which seven tRNA, four rRNA and seven protein-coding genes are duplicated in the IR. Sixteen genes contain a single intron, and only two genes have two introns. Phylogenetic analysis results strongly supported that *N.debneyi* was closely related to *Nicotiana sylvestris* and *Nicotiana tabacum*.

*Nicotiana* has been used as an important model plant in life science research, from around evolution, cytogenetics, interspecific hybridization and medical drug development (Winter 2000). It contains more than eighty species around the world, mainly distributed in the Americas, Australia and some species in Africa. *Nicotiana debneyi* is a tetraploid (2n = 4x = 48) wild tobacco native to the eastern coast of Australia classified in the section *Suaveolentes*. Resistances to black root rot, and blue mold and powdery mildew have been introduced from this species into *Nicotiana tabacum*, and the cross-compatible with *N.tabacu*m is cytoplasmic male-sterile (Japan Tobacco Inc 1994).

The chloroplast genome sequence has unique characteristics, such as uniparental inheritance, conserved sequence composition in a typical quadripartite genome structure, numerous variable sites, and coding regions (Bi et al. 2018). Chloroplast DNA sequence data are a versatile tool for plant identification or barcoding and to enhance our understanding about phylogeny of plant species. Studies on the chloroplast genome could also contribute useful information for our understanding of the molecular biology, physiology, and evolutionary of chloroplasts. However, the plastid genome of *N.debneyi* has not been reported. Herein, we report the complete chloroplast sequence of *N.debneyi* based on the next-generation sequencing and the annotated genomic sequence was deposited in the GenBank with the accession number MT985319, in order to provide resources for further genomic studies and chloroplast engineering.

Extraction of high-quality DNA was performed from 10 g of fresh leaves collected in the tobacco research station of Yunnan Academy of Tobacco Agricultural Sciences, Yuxi City, Yunnan province, China (N24°21′25.75″; E102°32′37.39″, voucher TW37), according to CTAB method (Doyle and Doyle 1987). Illumina 2 × 150 bp paired-end libraries were constructed with the NexteraXT DNA Library Preparation Kit (Illumina Inc., San Diego, CA) followed by sequencing on a Illumina Novaseq 6000 platform (Total Genomics Solution Limited, SZHT). Raw reads were filtered with Trimmomatic (Bolger et al. 2014), and de novo assembly of the plastid genome was carried out with the SPAdes-3.11.0 (http://cab.spbu.ru/software/spades/), using a k-mer set of 93,95,97,103,105,107,115 (Bankevich et al. 2012). The assembled chloroplast genome was annotated by Geneious v 9.0.2 (Kearse et al. 2012), followed by manual adjustments with the reference genome of *Nicotiana sylvestris* (GenBank: BK010741).

The complete chloroplast genome of *N.debneyi* (GenBank:MT985319) has a total length of 156,073 bp, which contains a pair of inverted repeats separated by small and large single copies. The genome consists of 129 genes and has 38.4% overall GC content. The genes can be classified into 37 tRNA, eight rRNA and 84 protein-coding genes, of which seven tRNA, four rRNA and seven protein-coding genes are duplicated in the IR. The four junctions between the single-copy segments and the inverted repeats were confirmed using PCR-based product sequencing. The results showed that the preliminary chloroplast genome was correctly assembled.

Twenty-five plastomes were aligned by MAFFT v.7.471 (Katoh et al. 2019) with default parameters. The best scoring Maximum Likelihood phylogenetic tree was calculated under GTR-GAMMA with 1000 bootstrap replicates using the program RAxMLv8.0 (Stamatakis 2014), including 20 representative species of Solanaceae and five outgroup species (Figure 1). The phylogenetic tree strongly indicated that *N.debneyi* formed a monophyletic clade and was closely related to two species (*Nicotiana sylvestris* and *Nicotiana tabacum*) by a high bootstrap value. The complete plastome can be subsequently utilized for genetic diversity and taxonomic studies of the genus Nicotiana.

**Figure 1.**
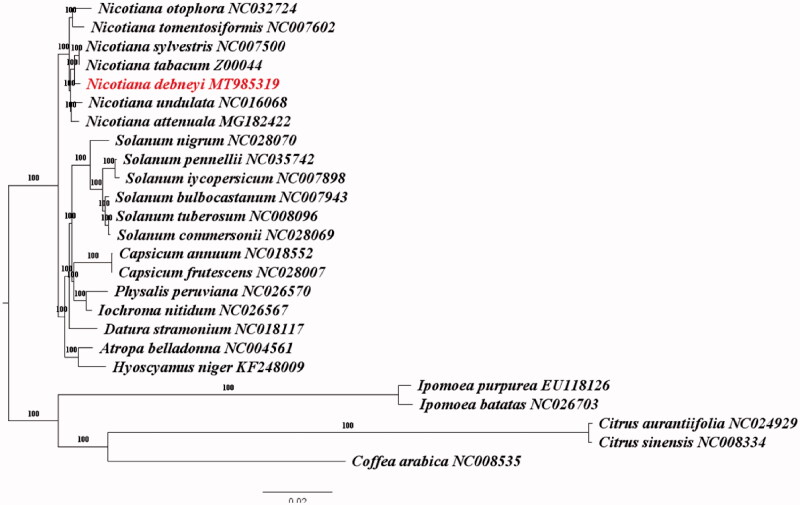
Phylogenetic relationships among 25 complete chloroplast genomes including 20 representative species of *Solanaceae* and five outgroup species. Bootstrap support values are given at the nodes.

## Data Availability

The genome sequence data that support the findings of this study are openly available in GenBank of NCBI at (https://www.ncbi.nlm.nih.gov/) under the accession no. MT985319. The associated BioProject, SRA, and Bio-Sample numbers are PRJNA690194, SUB8850676, and SAMN17245913 respectively.
